# New Challenges in Systems Biology: Understanding the Holobiont

**DOI:** 10.3389/fphys.2021.662878

**Published:** 2021-03-26

**Authors:** Maximino Aldana, Raina Robeva

**Affiliations:** ^1^Instituto de Ciencias Físicas, Universidad Nacional Autónoma de México, México, Mexico; ^2^Centro de Ciencias de la Complejidad, Universidad Nacional Autónoma de México, México, Mexico; ^3^Department of Mathematics, Randolph-Macon College, Ashland, VA, United States

**Keywords:** systems biology, holobiont, evolution, complex ecosystem, dynamics, challenge

More than a century ago, C. Darwin formulated the Theory of Evolution based on Natural Selection without knowing how information is transmitted from one generation to another. In Darwin's theory, the concept of *individual* is of fundamental importance: Individuals mutate, and advantageous mutations that improve adaptation to the environment are passed on to the next generations, perpetuating an improvement cycle. Now we know that mutations occur in the genomes of single individuals, and those mutations, if beneficial (or neutral), can be passed on to the next generations. However, in the Theory of Evolution formulated by Darwin and accepted for more than a century, the concept of individual is taken for granted: an individual is a single entity that contains (in its genome) all the necessary information to generate organisms with similar (or identical) phenotypes. However, the very concept of “individual,” on which Darwin's theory relies, has recently been challenged (Bordenstein and Theis, 2015; Suárez and Stenzel, 2020).

Today, we know that every multicellular organism is an ecological system. When such organisms emerged on Earth several millions of years ago, the planet was already crowded with microbial life. Consequently, the emergence and evolution of plants and animals were not only carried out in the presence of microbes but, in many cases, such evolution was only possible because of these microorganisms (Zilber-Rosenberg and Rosenberg, 2008; Alegado et al., 2012; Carpenter, 2012; Gaulke et al., 2016). Recent studies have unequivocally shown that there is a great variety of microbes living in plant and animal hosts, the totality of which is known as the host's *microbiota*. For humans, it has been estimated that the number of bacterial cells inhabiting a human body is comparable with the number of body cells (Abbot, 2016; Sender et al., 2016), and that the microbiota strongly interacts with its host, regulating important metabolic functions at all levels, including the genetic level (Bates et al., 2006; Wang et al., 2018).

The human microbiota helps in the development of the immune system, the fortification of bones, the digestion of food, the regeneration of skin, and affects many other essential metabolic functions. Furthermore, there is a strong correlation between the microbiota's composition and the occurrence of complex diseases such as obesity, diabetes, cancer, metabolic syndrome, inflammatory bowel disease, allergies to gluten and lactose, and even cognitive and neurological disorders such as autism and schizophrenia (Wang et al., 2017, 2018; Fan and Pedersen, 2021). Multicellular organisms have coevolved with microbes and strongly depend on them (even bacteria have their microbiota, which consists of viruses). Therefore, the question arises: what is an individual?

To emphasize that every individual is, in fact, a complex ecosystem, Rosenberg and his collaborators (Theis et al., 2016; Roughgarden et al., 2018) have proposed to use the term *holobiont* [first introduced by Lynn Margulis in a different context (Guerrero et al., 2013)] to refer to the host organism together with its microbiota. The holobiont is more than just an aggregate of cells that live together, as all cells in an organism carry the same genetic information in all organs. By contrast, in a holobiont, cells with different genetic compositions (even from different kingdoms), live together, interact, and exhibit complex dynamical behaviors. Imbalances in the holobiont ecosystem may lead to *dysbiosis*, a medical condition that may cause unwanted symptoms and, as mentioned above, has been associated with the onset of serious diseases. For a long time, predisposition to such diseases was attributed only to particularities in the genetic material of the host organism. However, if the microbiota's composition is strongly correlated with a complex disease, say obesity, it is possible that the microbiota that favors the occurrence of that disease is being transmitted from mother to offspring (Veigl et al., 2019). If that were the case, then the inheritance of phenotypes could be attributed not only to the genetic composition of the host organism but also to the transmission of microbes across generations.

Such connections would potentially challenge one of the central dogmas in the current Theory of Evolution, making Lamark's type of inheritance possible (Rosenberg et al., 2009). Indeed, there is strong evidence suggesting that. For instance, we know that obesity, diabetes, or breast cancer are inherited with high probability, as shown by familial studies. However, genetic markers for these diseases are yet to be discovered (a problem known as “*the missing heritability*”) (Manolio et al., 2009; Eichler et al., 2010; Génin, 2019). One possible explanation may hide in plain sight: our efforts to answer these questions are focused on looking for genetic markers only in the human genome (namely just in a tree of the entire forest), while we now know that the human microbiota is strongly correlated with the emergence of complex diseases. Therefore, when looking for genetic signatures of these diseases we should be looking at the entire forest; that is, consider not only the human genome but also the totality of genomes of all organisms in the microbiota—the *microbiome* (Sandoval-Motta et al., 2017).

Treatments for some illnesses, like the irritable bowel syndrome, that aim to rebalance the microbiota by transplanting fecal matter from a healthy person to a sick person (therapy known as “fecal transplant” or “bacteriotherapy”) date back to ancient China (De Groot et al., 2017). More recent studies (Woodworth et al., 2019) have shown that this approach is effective in reducing intestinal colonization with antibiotic-resistant bacteria, even though we may not yet fully understand the mechanism at the molecular or genetic levels. This suggests that transplanting bacteria from healthy to sick people may also work for other complex diseases such as obesity, cancer, or autism. The answer is yet to be discovered.

The strong symbiotic interactions between bacterial communities and their hosts (including humans) within the holobiont, were discovered only recently with the development of high-throughput sequencing techniques (Visscher et al., 2017). Most bacteria in the microbiota cannot be cultivated outside their host organism—let by themselves, these bacterial communities establish interactions dominated by competition, resulting in the dominance of one bacterial strain. It is the host that regulates these otherwise competing interactions, allowing different bacterial communities to inhabit the same organism (Foster and Bell, 2012; Coyte et al., 2015). As already mentioned above, bacteria in turn help the host in carrying out many different metabolic functions. High-throughput sequencing techniques provide a way to reveal the structure of complex ecosystems hidden inside the host and unveil those symbiotic interactions (Wang and Jia, 2016). The host, in turn, influences the composition of the microbiota. In the case of humans, the type of food we eat exerts an influence. A concrete example can be found in some bacteria living in the gut which transform carbohydrates into serotonin, a neurotransmitter associated with happiness (Stasi et al., 2019). Therefore, eating carbohydrates makes us happy, which creates a positive feed-forward cycle between us and gut bacteria that can lead to obesity and diabetes (It is still a matter of debate how the serotonin produced in the gut can cross the blood-brain barrier and reach the neurons in the brain).

It has been almost a decade since the pioneering work by Turnbaugh and his collaborators, who demonstrated that the bacteria in the human gut can determine important phenotypic traits (obesity) in mice (Turnbaugh et al., 2006). Since then, we have learned a lot about the symbiotic relationships between microbes and multicellular organisms. Mathematical models have also been deployed to investigate holobiont selection as an evolutionary force (Huitzil et al., 2018; Roughgarden, 2019). But we have only scratched the surface. There are many problems and questions that remain unsolved. In our opinion, one of the greatest challenges that we face for the twenty-first century regarding Systems Biology is to develop mathematical and computational models that help us understand the holobiont as a complex ecosystem. How and why such strong symbiotic relationships between bacteria and multicellular organisms have appeared throughout evolution? Did they appear just because it was possible? If not, what evolutionary advantages emerge from these symbiotic interactions? Will the concept of “individual” in evolutionary theories need to be reformulated (or replaced) to take into account the holobiont as a complex ecosystem subject to selection? What complex diseases could be cured by altering the microbiota's composition of the patient? Are there phenotypes that can be “inherited” through the microbiota and not through parental DNA? Is it possible to engineer a “healthy microbiota” or a microbiota that favors the emergence of desired phenotypes in the host organism?

The list of major challenges and unanswered questions is already long. And as we unravel some of these questions in the future, the list will get even longer. The Systems Biology community around the world faces a significant challenge—we need experimental methods, mathematical models, and computational approaches that combine the best available data from genomics, metabolomics, and proteomics with existing knowledge in the life sciences to help us understand the evolution, dynamics, and behavior of holobionts not as individuals, but as complex ecosystems.

## Author Contributions

All authors listed have made a substantial, direct and intellectual contribution to the work, and approved it for publication.

## Conflict of Interest

The authors declare that the research was conducted in the absence of any commercial or financial relationships that could be construed as a potential conflict of interest.
